# Characterization of TEMPO-Oxidized Cellulose Nanofiber From Biowaste and Its Influence on Molecular Behavior of Fluorescent Rhodamine B Dye in Aqueous Suspensions

**DOI:** 10.1007/s10895-024-03824-4

**Published:** 2024-07-01

**Authors:** Mehmet Kaya

**Affiliations:** Faculty of Science, Department of Chemistry, Ataturk University, Erzurum, 25240 Turkey

**Keywords:** Cellulose nanofiber, TEMPO oxidation, Dye aggregation, Bio-waste, Photophysics

## Abstract

Cellulose nanofiber (CNFs) obtained through TEMPO oxidation was structurally characterized using FT-IR (Fourier Transformed Infrared) and SEM (Scanning Electron Microscopy) spectroscopy. The molecular aggregation and spectroscopic properties of Rhodamine B (Rh-B) in CNFs suspension were investigated using molecular absorption and steady-state fluorescence spectroscopy techniques. The interaction between CNFs particles in the aqueous suspension and the cationic dye compound was examined in comparison to its behavior in deionized water. This interaction led to significant changes in the spectral features of Rh-B, resulting in an increase in the presence of H-dimer and H-aggregate in CNFs suspension. The H-type aggregates of Rh-B in CNFs suspensions were defined by the observation of a blue-shifted absorption band compared to that of the monomer. Even at diluted dye concentrations, the formation of Rh-B’s H-aggregate was observed in CNFs suspension. The pronounced aggregation in suspensions originated from the strong interaction between negatively charged carboxylate ions and the dye. The aggregation behavior was discussed with deconvoluted absorption spectra. Fluorescence spectroscopy studies revealed a significant reduction in the fluorescence intensity of the dye in CNFs suspension due to H-aggregates. Furthermore, the presence of H-aggregates in the suspensions caused a decrease in the quantum yield of Rh-B compared to that in deionized water.

## Introduction

Due to their biodegradability, non-toxicity, and numerous other attractive features, such as, cellulose, chitosan, and starch-based natural materials have become the focus of scientists’ investigations. Cellulose, in particular, being the most abundant natural material in the world, possesses various properties such as workability and the ability to be isolate at nano-sized dimensions, thanks to its excellent biological structure [1]. It is well-known that materials originating from cellulose-based nanofibers offer significant advantages in terms of applicability and developability [2–9]. In recent years, cellulose nanofibers (CNFs) containing both amorphous and crystalline regions have gained widespread usage owing to these unique properties. CNFs are thin-sized and high-strength materials that can be obtained mechanically or chemically from wood-derived fibers [10–13]. Their superior optical and mechanical properties can be tailored for specific applications, leading to an accelerated exploration of such nanostructures. These remarkable characteristics endow the nanofibers with great potential in the material world. The hydrogen bonds in their structures hold the cellulose chains together through strong interactions, which include [14, 15], making them promising materials for use in a wide range of fields, including electronic and optical devices [16], barriers [17], foods [18], pharmaceutical industry [19] and cosmetics [20].

Over the past years, numerous studies have emerged concerning the interaction of cellulose with dyes due to its outstanding properties. These studies cover various applications, such as photocatalytic effects, dye removal, composite film production, and textile dyeing [21–24]. There are also several studies on the aggregation behaviour of Rh-B [25, 26]. However, detailed studies on the photophysical properties and aggregation behavior of dyes in suspension or solution, especially their interactions with CNFs, are thought to be lacking. Thus, considering their potential applications in optoelectronics [27, 28], sensing [29, 30], and imaging [31, 32], the spectral features of dyes interacting with CNFs are noteworthy. When cellulose nanofibers are suspended in a solvent, they allow dyes to penetrate into their micro and/or nanopores [33]. This leads to the arrangement of cellulose-dye or cellulose-dye-cellulose hydrogen bond chains due to the solvent effect. As a result, it is anticipated that by modifying the spectral properties of dye molecules trapped in cellulose pores, the aggregation behavior in the ground and excited states can be controlled at the desired electromagnetic wavelength. Therefore, the effect of CNFs-based materials on fluorescence dyes presents a very interesting phenomenon.

In our various studies, we have revealed the photophysical behavior of dyes with strong fluorescence properties, such as Rhodamine B (Rh-B), Merocyanine540 (MC-540), Pyronin B, and Pyronin Y, in several media [34–42]. Additionally, the general properties of cellulose materials and the production of cellulose aerogel from tea stem wastes (TSW) were described in previous studies [43, 44]. Some of the dyes are employed in dye lasers to regulate the wavelength of electro-optical magnetic radiation, in photo dynamic therapy, in the colouring of numerous materials, in the enhancement of the surface properties of certain materials and in a multitude of other fields. The behaviour of dyestuffs, particularly in diverse media containing substances such as nanosized particles, can be modified. This offers superior advantages to the dyestuffs in terms of rendering them suitable for use in desired areas. The objective of this study is to elucidate how the characteristics of a colourant, such as rhodamine B (Rh-B), which can be employed in a multitude of applications, alter as a consequence of its interaction with (CNFs) and the advantages it confers. In this study, we used cellulose isolated from a natural waste source to produce and characterize 2,2,6,6-tetramethylpiperidine (TEMPO)-oxidized CNFs material using spectroscopic methods (FTIR, Fourier Transformed Infrared Spectroscopy, and SEM, Scanning Electron Microscopy). Then, investigated the effect of produced CNFs on the aggregation behavior of Rhodamine B (Rh-B) fluorescent dye was investigated. The tempo-mediated oxidation method is a typical approach for preparing nanocellulose. Through this method, the carboxyl groups on the cellulose surface are increased, and the cellulose particles are better dissolved in the aqueous phase. The carboxylate anions in the cellulose nanofibril cell wall are expected to exhibit strong interactions with positively charged compounds [45], and we found that Rh-B, which has a positive charge center in an aqueous medium, showed strong interactions with TEMPO-oxidized CNFs. Leveraging these CNFs particle properties, their effect on the molecular behavior of Rh-B using various spectroscopic techniques were investigated. By means of UV-Vis absorption and fluorescence spectroscopy, we obtained corrected absorption and fluorescence spectra of the dyes, which had penetrated the natural nanofiber polymer chains in the CNFs/deionized water suspensions. Moreover, we calculated the fluorescence quantum yields for Rh-B, as well as the average particle size and zeta potential of the CNFs suspensions, taking ambient conditions into account.

## Experimental Section

### Materials

**Fig. 1:**
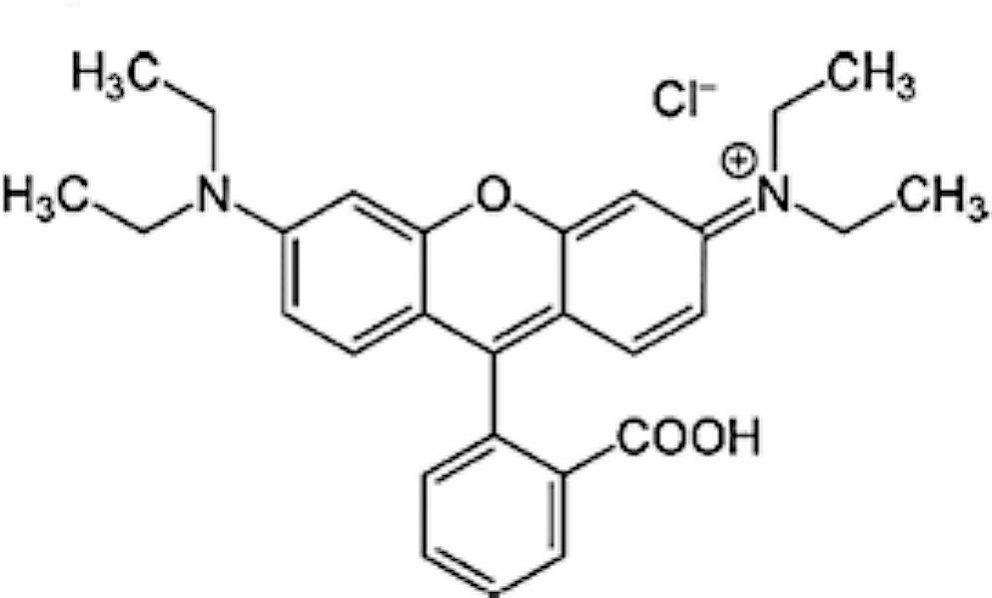
Chemical structure of Rhodamine B

Pure cellulose was isolated from tea stem wastes (TSW) and isolation process reported previously [43]. Rh-B (b) dye which is purchased from Merck, was used without further purification and its chemical formula were presented in Fig. 1. Sodium hydroxide-NaOH, hydrochloric acid-HCl, (reagent grade, Sigma-Aldrich), sodium hypochlorite-NaClO, 2,2,6,6-tetramethylpiperidine (TEMPO, 99.9%, Sigma-Aldrich), sodium bromide (NaBr, 99.6%, Sigma-Aldrich) were used as received without further modification.

### CNFs Production

Cellulose samples isolated from agricultural biowastes were used for preparation of CNFs. Typically, TEMPO oxidized CNFs were prepared according to the procedures reported by Follain et al., Si et al., and Taira et al. [46–48]. Cellulose samples (1 g) were mixed with 0.1 mmol TEMPO and 1.0 mmol NaBr in 100 ml distilled water to obtain a suspension. Then, 12% NaClO solution was adjusted to pH 10 using 0.1 M HCl and 0.5 M NaOH solutions. TEMPO mediated oxidation was initiated by adding the desired amount of NaClO solution (1.3-5.0 mmol NaClO per gram of cellulose) and the reaction was continued by stirring at 700 rpm at room temperature. The suspension was then dialyzed against distillated water for three days to remove excess base or acid. The TEMPO-oxidized cellulose was thoroughly washed with water by filtration and stored at 4 °C before further processing or analysis. The obtained CNFs were diluted to 1.0 wt%. The suspensions were stored at room temperature until further use and characterization.

### Preparation of the dye-CNFs Suspensions

**Fig. 2:**
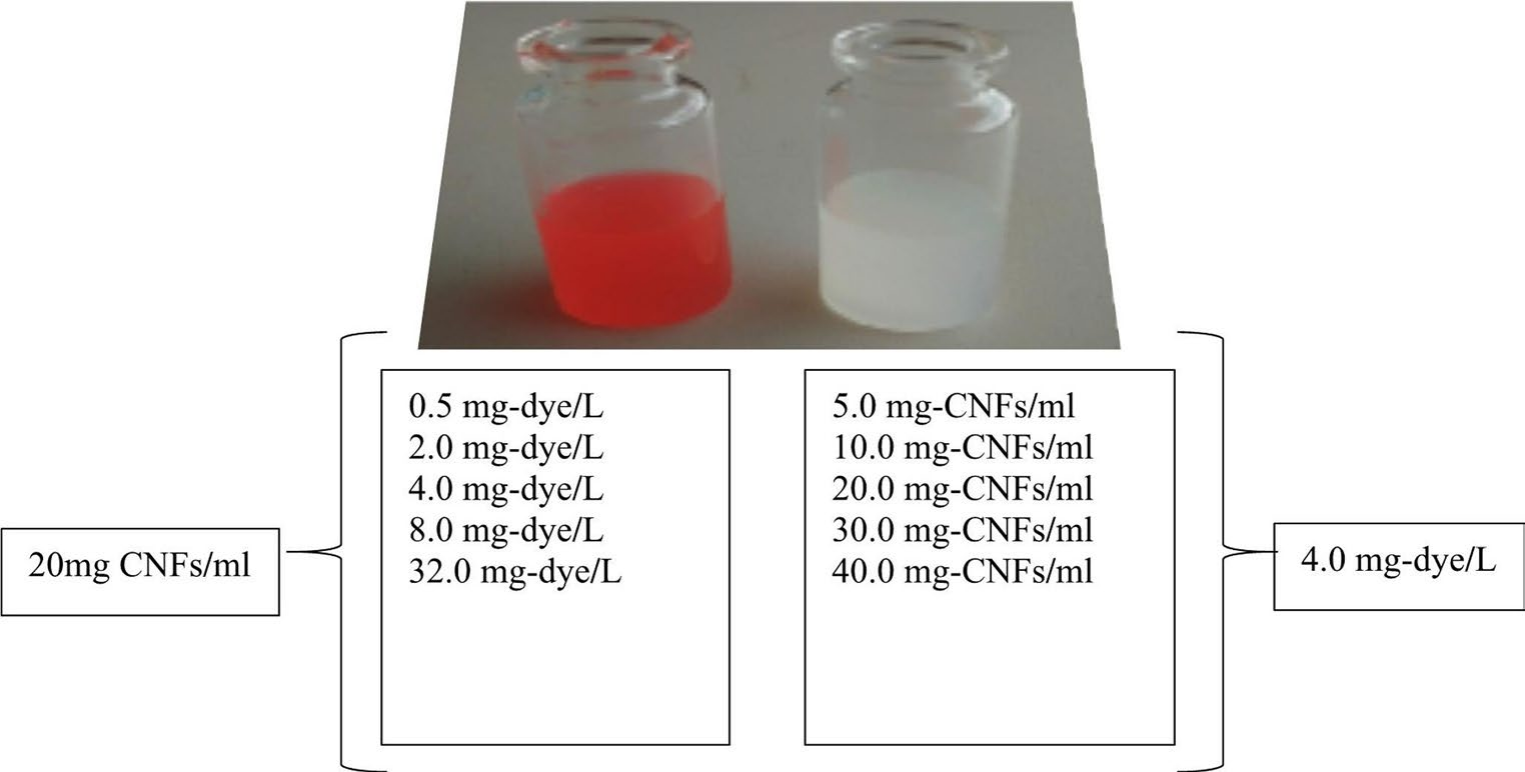
CNFs suspension ratios

CNFs suspensions and dye solutions were prepared in deionized distilled water. Absorption and emission studies were performed in different suspensions of CNFs at a constant dye concentration and at different dye concentrations versus a constant suspension ratio of CNFs. The suspension ratios of CNFs used were 5.0, 10.0, 20.0, 30.0 and 40.0 mg/ml while the dye concentrations used were 0.5, 2.0, 4.0, 8.0 and 32 mg/L. 4.0 mg-dye/L and 20.0 mg-CNFs/ml were chosen for fixed dye concentration and CNFs suspension ratio (Fig. 2).

### Degree of Oxidation (DO)

The degree of oxidation of hydroxymethyl groups (DO) was expressed as a ratio between the amount of oxidized hydroxymethyl groups and the total hydroxymethyl groups, which was determined by conductimetric titration and DO, as determined by conductimetry titration, reached a value of 87.0%. A 100-mg cellulose sample was suspended or dissolved in 30 mL of a 0.01 mol/L hydrochloric acid solution. Following a 15-minute period of stirring, the suspension was titrated with a 0.005 mol/L NaOH solution under stirring. Conductivity was monitored throughout the titration process using a conductivity meter. The titration was terminated when the pH reached 11–12. The dissolved oxygen (DO) concentration was calculated using the following equation: [49]

$$\text{DO}=\frac{162\times C\times (V_{2}-V_{1})}{m-36 \times C\times (V_{2}-V_{1})}\times 100 \%$$

### Characterization

UV–Vis absorption and fluorescence spectra for all samples (in quartz cuvette) were recorded at room temperature with Perkin Elmer Lambda 35 UV/VIS Spectrophotometer and Agilent Cary Eclipse G9800A spectrofluorophotometer, respectively Fluorescence quantum yields were calculated by using “Parker-Rees Method”. The equation for this method is given below;

1 $${\varnothing }_{s}={\varnothing }_{r}\left(\frac{{D}_{s}}{{D}_{r}}\right)\left(\frac{{{\upeta }}_{s}^{2}}{{{\upeta }}_{r}^{2}}\right)\left[\left(\frac{1-{10}^{{-OD}_{r}}}{1-{10}^{{-OD}_{s}}}\right)\right]$$

where ‘$\varnothing$’ is the fluorescence quantum yield, ‘D’ is the integrated area under the corrected fluorescence spectrum, ‘η’ is the refractive index of the solution, and ‘OD’ is the optical density at the excitation wavelength (λex = 390 nm). The subscripts ‘s’ and ‘r’ refer to the sample and reference solutions, respectively. FTIR spectra were recorded in the region of 4000 − 650 cm^-1^ on a Spectrum-100 FTIR spectrometer at a resolution of 4 cm^-1^. SEM analysis was carried out using JEOL JSM 6610 instrument equipped with EDS analyzer. Average particle size distribution and zeta potential of cellulose nanofibers were measured using the Zetasizer Nano ZSP (Malvern Instruments Ltd., Malvern, UK). When the zeta potential is strongly negative (more negative than − 30 mV) or strongly positive (more positive than + 30 mV), the electrostatic repulsion between particles becomes significant. This repulsion prevents the particles from coming close together and agglomerating, leading to a stable suspension.

## Results and Discussion

### FTIR Spectrum of CNFs

**Fig. 3:**
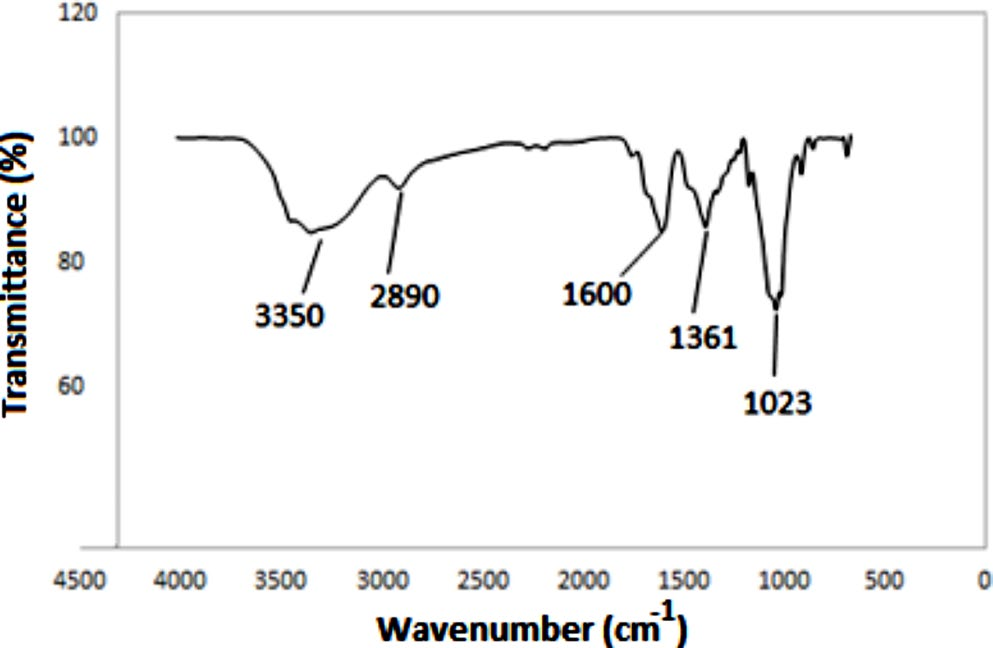
FTIR spectrum of CNFs

FTIR spectroscopy is a powerful analytical tool that enables the detection of new functional groups and the study of chemical structure changes resulting from various processes, including chemical and mechanical treatments. In this study, cellulose nanofibers underwent FTIR analysis to determine the functional groups present in their chemical structures. Figure 3 depicts the FTIR spectra of CNFs where the absorption peak at 1600 cm^− 1^ and 1361 cm^− 1^ can be observed indicating the present of the asymmetrical stretching vibration of carboxyl groups (–COO^−^). The characteristic absorption peaks of carboxyl groups can be observed in the FTIR spectra as symmetric stretching vibrations at 1600 cm^− 1^ and 1361 cm^− 1^ [50]. This presence of negatively charged -COO^−^ groups plays a significant role in promoting the efficient dispersion of CNFs in water via electrostatic repulsion [51]. The result indicates that the carboxyl group is indeed incorporated into CNFs after TEMPO oxidation. In addition to that, the peak at wavenumber 1023 cm^− 1^ also been observed attributing to the present of C-O-C groups. Significant vibration peaks are also clearly visible in the spectrum, corresponding to the C–H stretch (2890 cm^–1^) and O–H stretch (3350 cm^–1^) [52].

### Particle Size and Zeta Potential Analysis

Zeta potential is a crucial index representing the degree of repulsion between adjacent particles carrying the same charge in a colloidal system, which determines the stability of the colloidal dispersion. The higher the absolute value of the zeta potential, the better the stability of the colloidal dispersion. In other words, it indicates a higher ability for dissolution or dispersion rather than agglomeration [53]. Table 1 shows the zeta potential and particle size distribution of CNFs. As observed, it exhibits negative ξ (zeta potential) values peaking at -49.54mV. The increase in the absolute value of the zeta potential with the addition of NaClO suggests that the CNFs dispersion becomes more electrically stable. This is attributed to the higher NaClO content supporting oxidation, thereby converting numerous hydroxyl groups on the surface of primary CNFs into COO^−^. As a result, a strong electrostatic repulsion between microfibers in water contributes to the uniform distribution of CNFs in the aqueous solution. Combining chemical and mechanical treatments results in CNFs with the smallest average particle size diameter of 64.8 nm. When fibers are obtained through TEMPO oxidation, they become significantly finer compared to fibers processed solely by either chemical or mechanical methods. TEMPO oxidation assists in the dispersion of packed fibrils and enables easier fragmentation during microfluidization due to the application of high shear forces.

**Table 1:** Zeta potential and particle size of CNFs

Sample	Average Dimension (nm)	Zeta Potential (mV)
TEMPO Oxidized-CNFs	64.8	-49.54

### SEM Analysis of CNFs

**Fig. 4:**
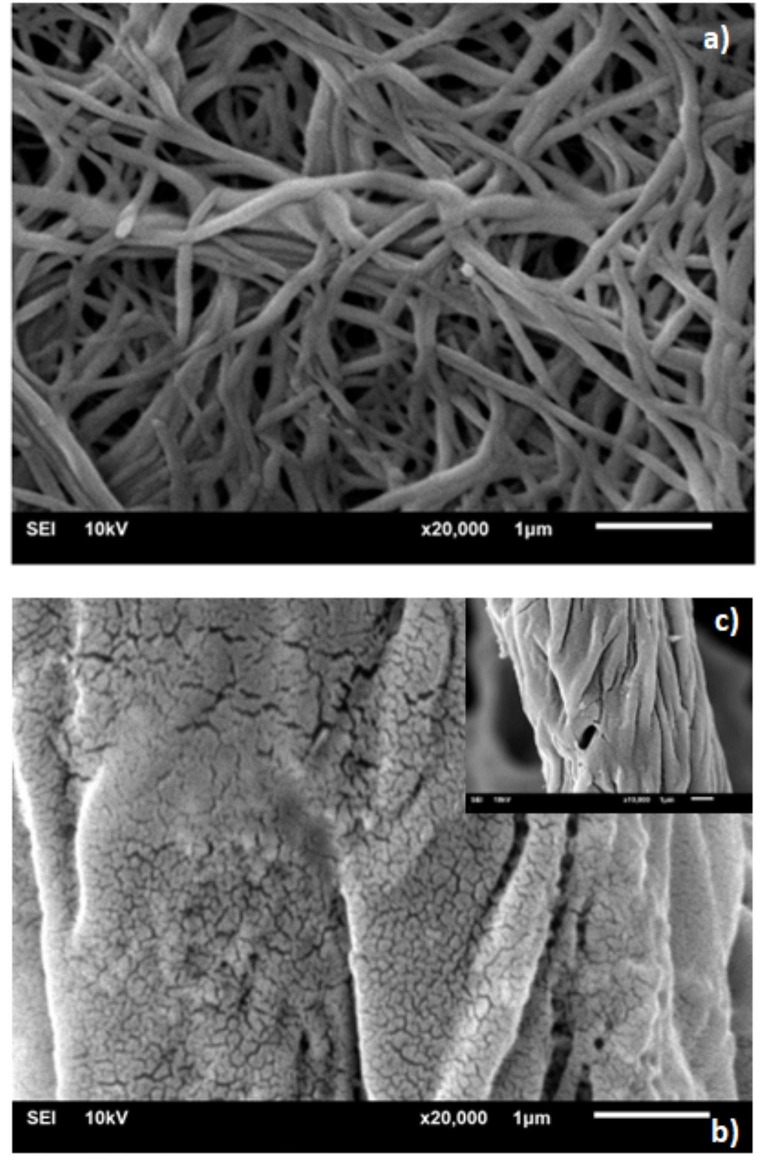
SEM image of CNFs, (**a**) CNFs structure and (**b**) raw cellulose structure under 1 μm magnification (20,000x). The picture inset (**c**) show the structure of raw cellulose under 1 μm magnification (10,000×)

From Fig. 4a, it is evident that CNFs are nano-sized and have a network structure when compared to raw cellulose (Fig. 4b). The particle size analysis indicates an average diameter of approximately 64 nm for the CNF fibers. The images show a random distribution of fiber chains forming clusters. TEMPO oxidation transforms the macrocellulose structure into nanofibers, leading to improved physicochemical properties of the material. Carboxylate anions introduced to cellulose during oxidation interact with positively charged rhodamine b (the positive charge on the quaternary ammonium group), affecting its photophysical behavior. The nanoscale size of the nanofibers in the suspension medium and the presence of carboxylic groups on their surfaces promote water retention within the structure, allowing the dye to penetrate the fibers more effectively. The following photophysical findings demonstrate that this strong interaction significantly influences the aggregation behavior of the dye.

### Absorption and Fluorescence Emission Spectra of CNFs-Rh-B Suspensions

**Fig. 5:**
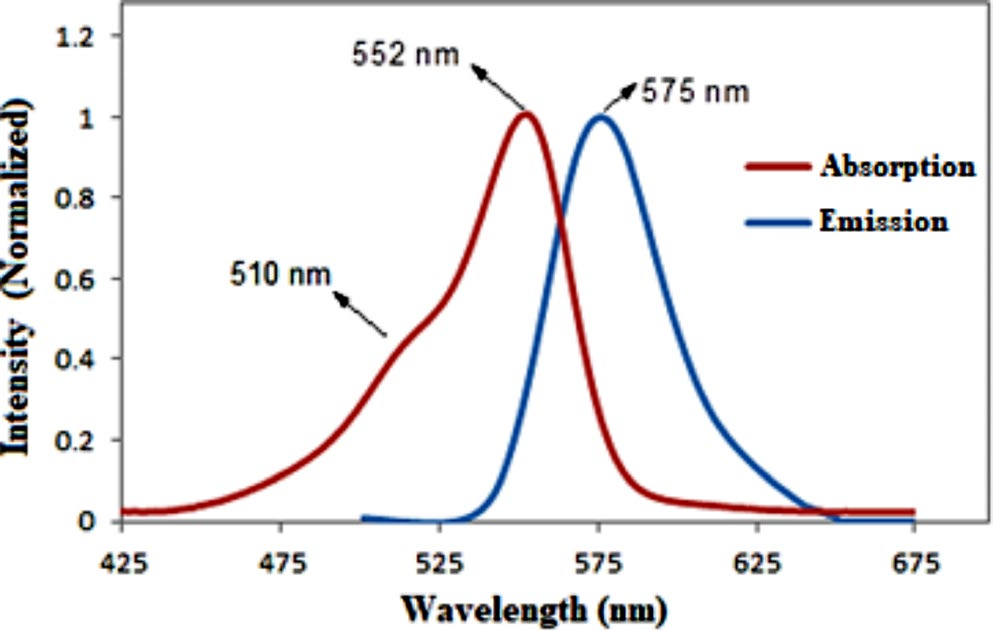
Normalized absorption and emission spectra of Rhodamine B (4.0 mg/l) in deionized water

Figure 5 shows two absorption bands in the Rh-B compound’s absorption spectrum taken in deionized water at a concentration of 4.0 mg/l. The band around 552 nm is known as the monomer band, while the weak intensity band around 510 nm is associated with a vibration band at low concentrations [54]. Aggregation bands, such as the H-aggregation band can be observed at around 510 nm, in which generally have a higher energy and shorter wavelength (H-aggregate) or lower energy and longer wavelength spectra (J-aggregate) compared to monomer bands [55]. Furthermore, the band around 575 nm corresponds to the fluorescence emission band maximum of the Rh-B molecule in pure water. A difference of approximately 23 nm (known as the Stokes shift) exists between the absorption and emission maxima. These observations offer valuable insights into the optical characteristics and behavior of the Rh-B compound in water at different concentrations, shedding light on its aggregation and emission properties.

**Scheme 1:**
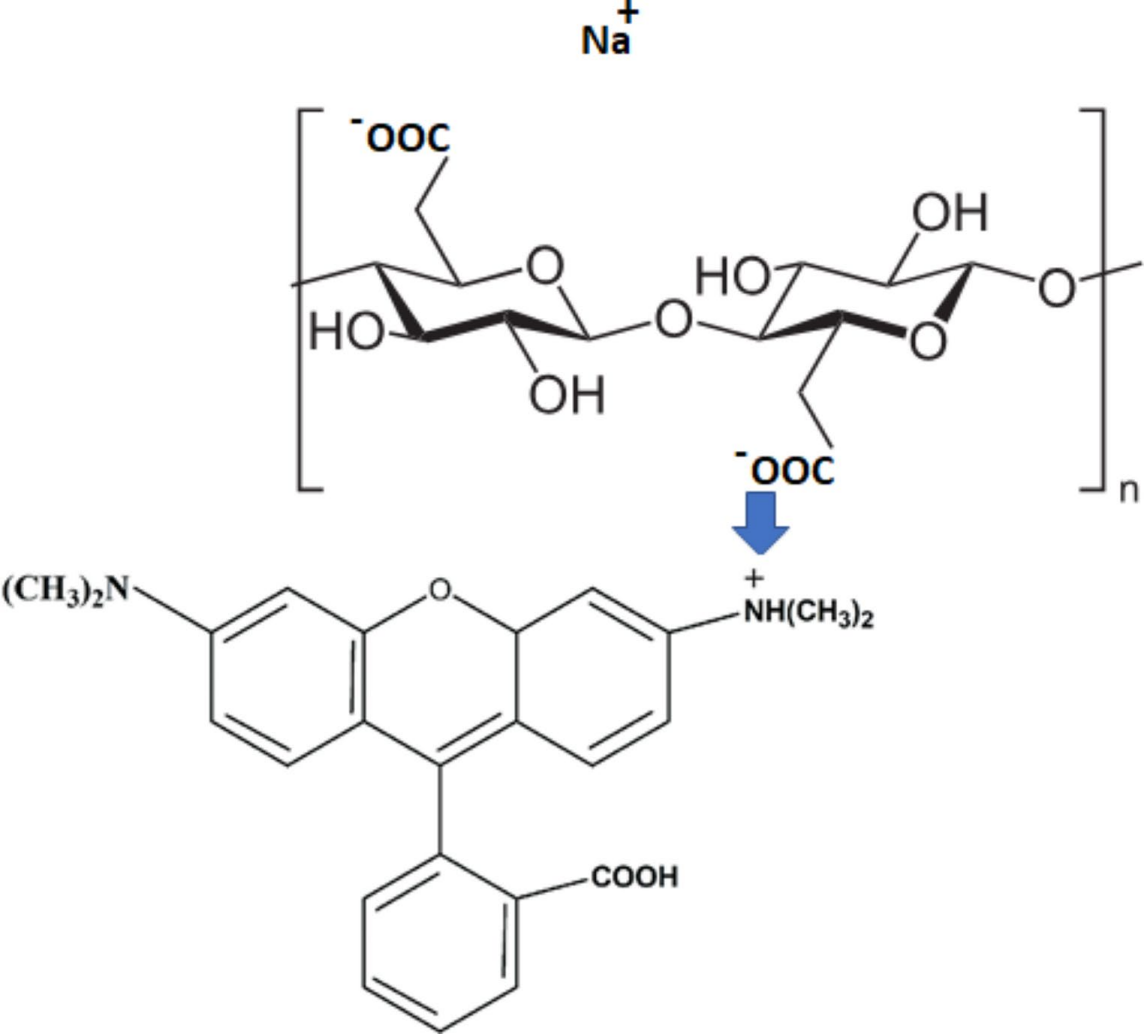
Interaction (Representative) of Rhodamine B and CNFs

**Fig. 6:**
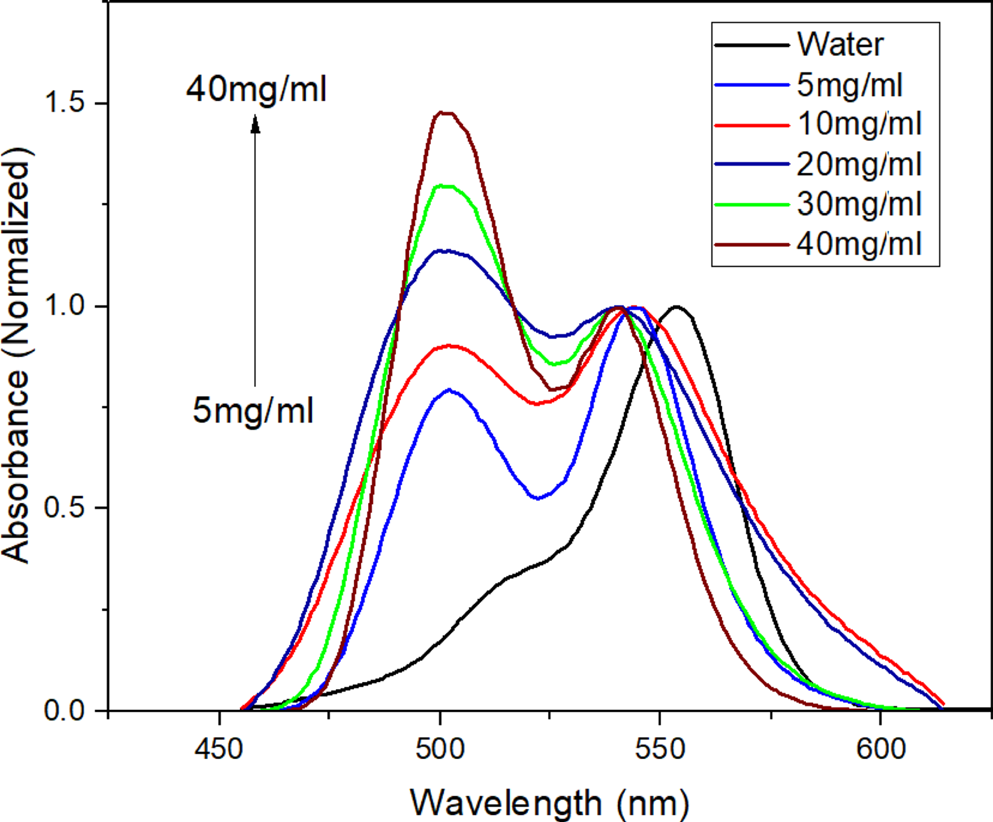
Normalized (monomer band) absorption spectra of Rhodamine B (4.0 mg.l^-1^) at different CNFs suspensions

Figure 6 clearly demonstrates significant changes in the spectral behavior of the Rh-B molecule during the absorption study conducted in CNFs suspensions at various ratios. Upon thorough scrutiny of the shape, it becomes evident that the aggregation intensity increases considerably with higher suspension ratios. Across all suspensions, the monomer band experiences a blue-shift of approximately 10 nm within this range. Notably, the H-dimer and H-aggregate bands show a substantial increase in intensity. This observation indicates that the Rh-B molecule, possessing a positive charge center, undergoes aggregation due to its interaction is exhibited in Scheme 1 as a representative) with the carboxylate anions present in cellulose nanofibers, as previously explained. Furthermore, as the CNFs ratio increases, the molecule interacts with a greater number of carboxylate anions, leading to a further increase in the aggregate bands’ intensity.

**Fig. 7:**
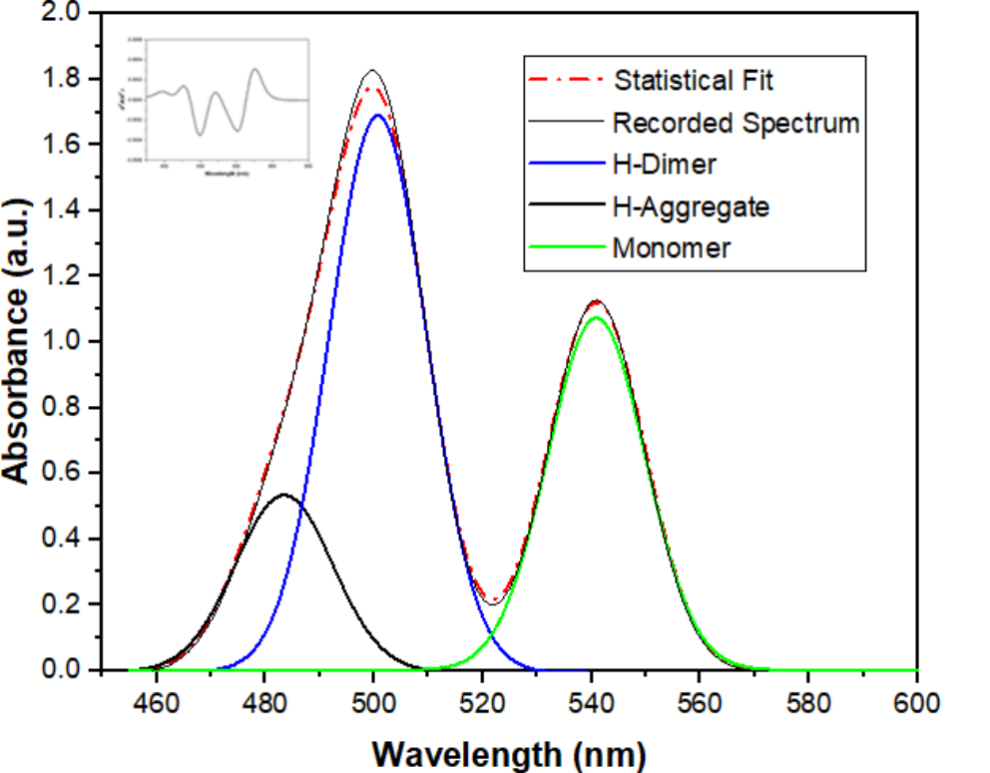
The deconvoluted spectra of Rhodamine B (32.0 mg.L^-1^) in CNFs suspension (40.0 mg.ml^-1^). The inset shows their second-derivative spectrum

The photophysical behavior of Rh-B compound at constant concentration (4.0 mg/l) at different CNFs suspension ratios (5.0, 10.0, 20.0, 30.0 and 40.0 mg/ml) was investigated. To explore the robust H-aggregation behavior of Rh-B in CNFs suspensions, the absorption spectrum of the aggregate type was subjected to component analysis. Upon careful examination, three distinct bands were clearly visible as shown in Fig. 7 where: the H-aggregate band is at approximately 483 nm, the H-dimer band at 500 nm, and the monomer band at 540 nm. The deconvolution spectra (Fig. 7) revealed severe blue-shifts in the band maxima. Besides, paying attention to the 2nd derivative spectrum of the absorption spectrum (on the top left) confirms the detection of three bands corresponding to the monomer, H-dimer, and H-aggregate types. Upon examination, the spectra obtained through fitting using the Gaussian function in Originlab Pro 2022 program, the pronounced severity of H-aggregation becomes evident. The concordance of the fitted signal with the spectra further confirms this hypothesis.

**Fig. 8:**
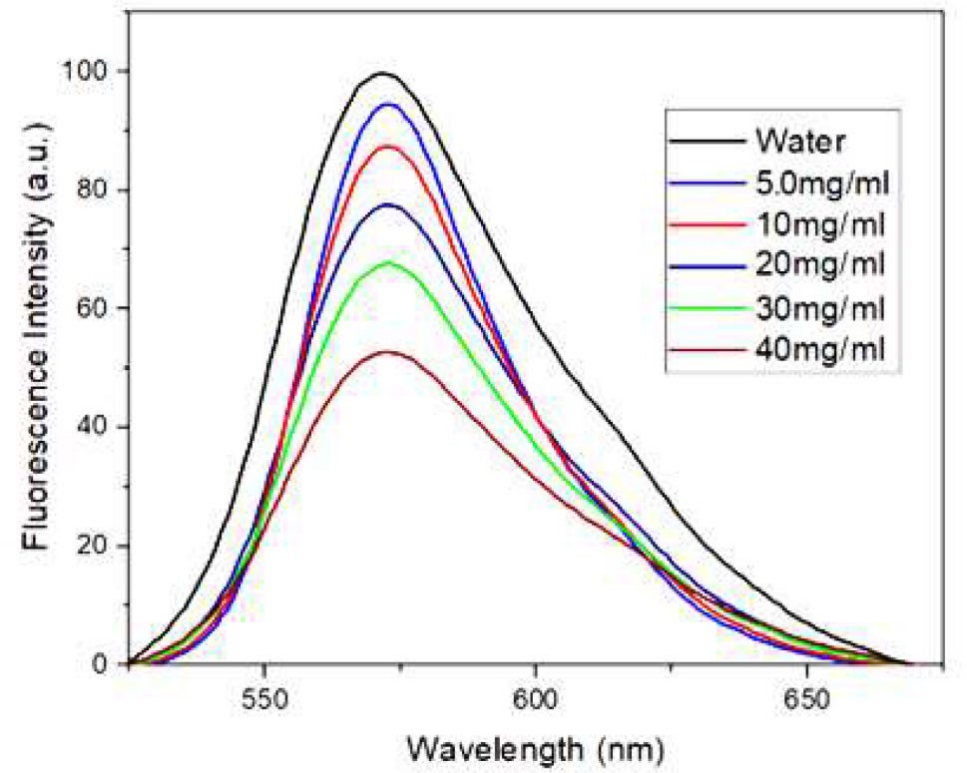
Emission spectra of Rhodamine B (4.0 mg.l^-1^) at different CNFs suspensions

**Fig. 9:**
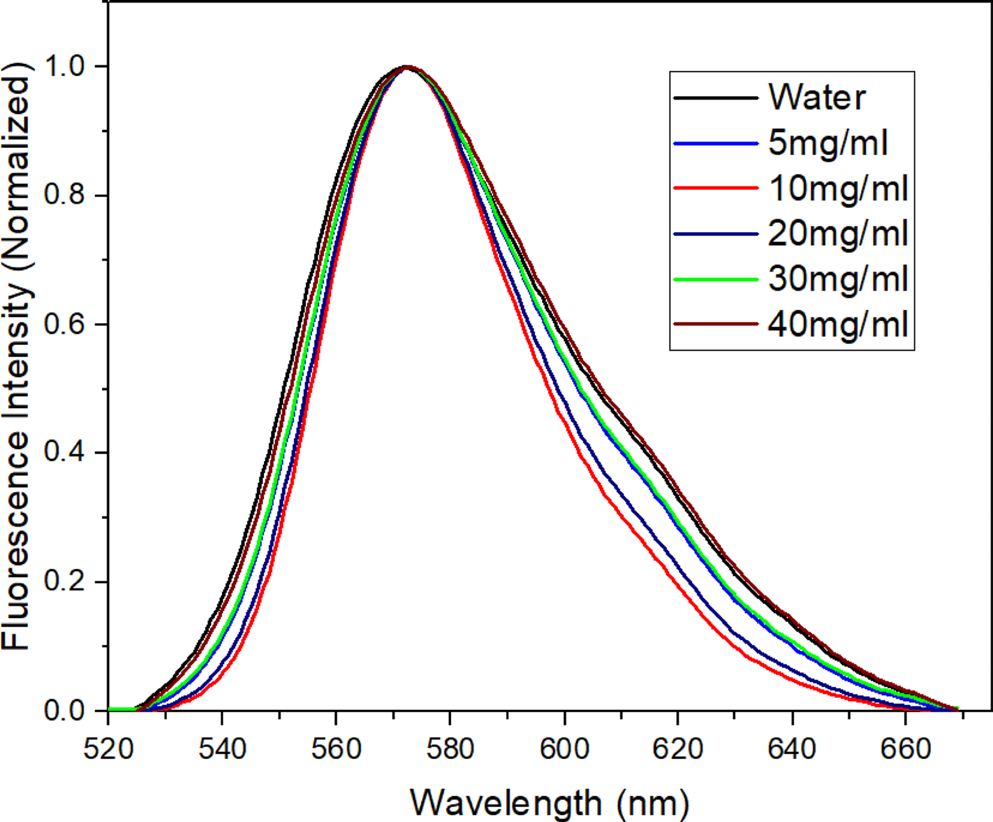
Normalized emission spectra of Rhodamine B (4.0 mg.l^-1^) at different CNFs suspensions

The fluorescence emission behaviour of the Rh-B molecule was also investigated in varying CNFs suspension ratios while keeping the Rh-B concentration constant. Upon reviewing Fig. 8, it is evident that an increase in the CNFs suspension ratio results in a slight decrease in fluorescence intensity despite the constant Rh-B concentration. Additionally, the fluorescence emission band in the CNFs suspension shifts slightly towards the blue (575→572 nm) compared to the deionized water environment, indicating an approximate 3 nm blue-shift. Unlike the absorption spectra, significant blue-shifts were not observed in the fluorescence spectra. Figure 9, shows the normalized emission spectra, which supports this observation. Although there is no significant shift in fluorescence bands, a substantial reduction in their intensities is clear. The pronounced decrease in fluorescence intensity in Fig. 8 is apparent. The decrease in fluorescence intensity, despite the increase in H-aggregate absorption intensity, can be attributed to the absence of fluorescence properties in the H-aggregates [56]. Furthermore, the presence of fluorescence spectra is associated with the fluorescence-active monomer type within the structure. However, as the CNFs suspension ratio increases, the H-aggregate type becomes dominant over the monomer. This may explain the observed decrease in fluorescence bands. An optimum concentration of 4.0 mg/L was selected for Rh-to study effects of CNFs quantity on quenching and aggregation, while minimizing spectral interferences due to concentration.

**Fig. 10:**
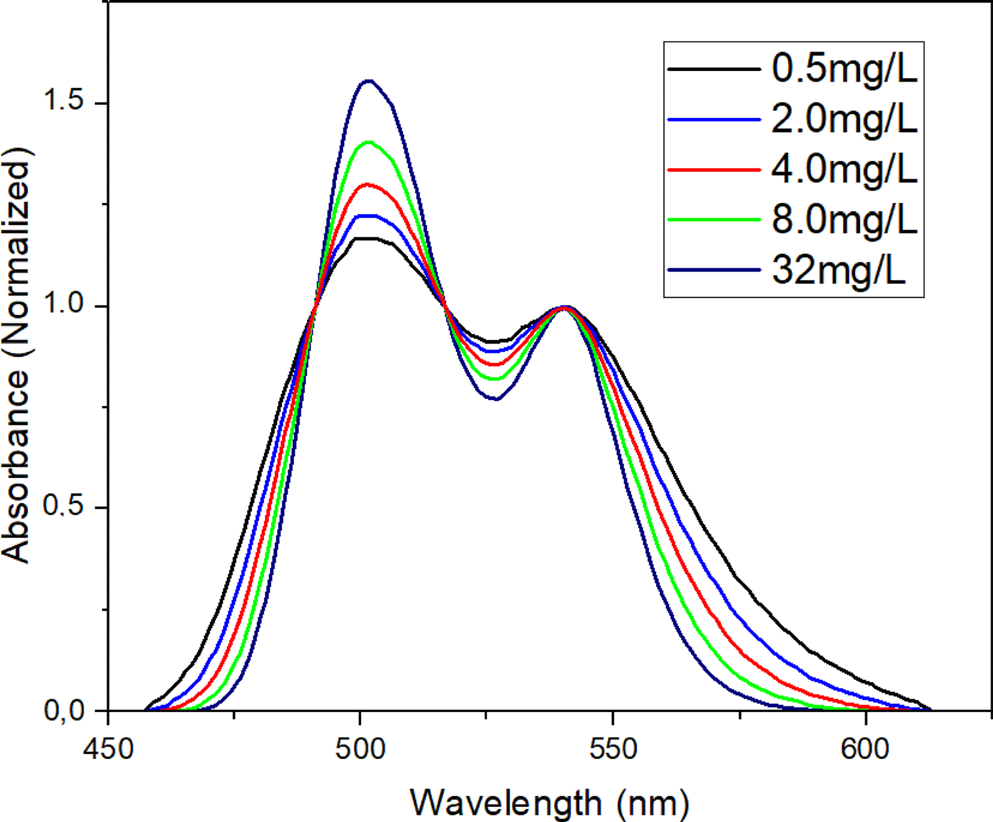
Normalized absorption spectra of Rhodamine B at different concentrations (CNFs = 20.0 mg.ml^-1^)

**Fig. 11:**
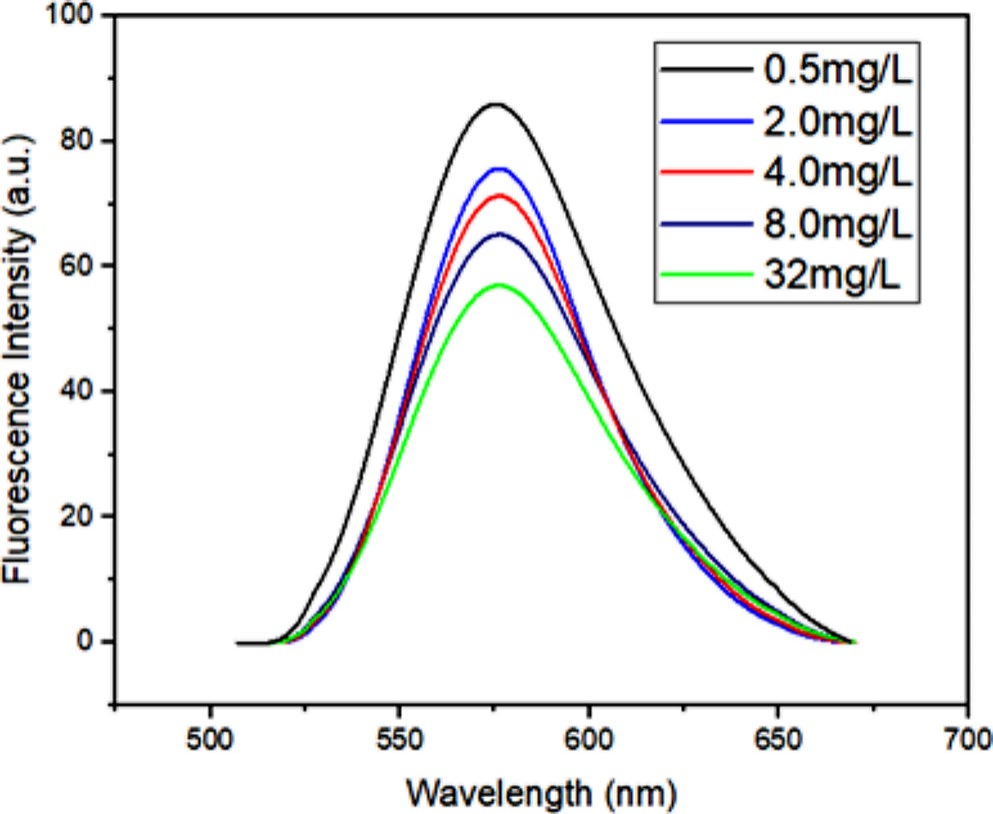
Emission spectra of Rhodamine B at different concentrations (CNFs = 20.0 mg.ml^-1^)

The molecular behavior of the Rh-B molecule’s absorption spectrum were investigated in a constant CNFs suspension ratio, Fig. 10 depicts the normalized absorption spectra of Rhodamine B at different concentrations, where, an increase in the dye concentration leads to an enhancement in the H-aggregation band intensity. This phenomenon is expected since the rise in dye concentration increases the electrostatic interactions and clustering of molecules within the suspension. However, in the context of a constant CNFs suspension ratio, the dye’s aggregation behavior and band maxima were not significantly affected by the self-effect. Likewise, in Fig. 11displays fluorescence emission spectra under the same environmental conditions, a similar spectral trend is observed. From the normalized emission spectra in Fig. 11, no significant spectral shift was observed. On the other hand, despite the increase in the Rh-B molecule’s concentration (0.5, 2.0, 4.0, 8.0 and 32.0 mg/l), a reduction in emission intensity is observed. This decrease in fluorescence intensity can be attributed to the absence of fluorescence properties in the H-aggregates, demonstrating a correlation with the increase in H-aggregation intensity. The results further provide valuable insights on the aggregation behavior and fluorescence characteristics of the Rh-B molecule in CNFs suspensions at varying dye concentrations, shedding light on the interactions and dynamics within the system.

### Fluorescence Quantum Yield of CNFs-Rh-B Suspension

The relative fluorescence quantum yield is a crucial photophysical parameter that indicates the effectiveness of a substance’s fluorescence properties. In this research, the fluorescence quantum yields (Φf) of samples containing dye and CNFs were determined by comparing them with Rhodamine 101 in ethanol as a reference solution with a quantum yield of 1.0 and the Parker-Rees method [57] was used to calculate quantum yields of Rh-B in CNF suspension. The results showed that the fluorescence quantum yield values for all suspensions were quite low (approximately Φ_f_ < 0.012) compared to the quantum yield of Rh-B in water (Φ_f_ 0.31 for Rh-B in water [58]). The significant decrease in fluorescence quantum yield can be attributed to the formation of H-aggregates of Rh-B in the CNFs suspension. As a consequence, the H-aggregates in the CNFs suspension of CNFs are non-fluorescent, leading to a very low fluorescence quantum efficiency in the system when compared to other reported studies.

## Conclusions

In this study, cellulose fibers obtained from a biowaste product were subjected to TEMPO oxidation, reducing them to nanosize. The structure of tempo-mediated cellulose nanofibers was elucidated by various spectroscopic methods. Carboxylate ions included in the cellulose structure with tempo oxidation showed a strong interaction with rhodamine b. The effect of this strong interaction on the photophysical properties of rhodamine b was characterized by UV-vis absorption and fluorescence spectroscopy. It was determined that rhodamine b showed a strong h-aggregation tendency in aqueous suspensions of cellulose nanofibers. It was revealed that the aggregate intensity was parallel to the increasing nanocellulose fiber suspension ratio. However, the intense aggregation of Rh-B was attributed to the strong interaction of positively charged Rh-B molecules with carboxylate anions in the medium because of TEMPO oxidation. With this interaction, it was determined that the dye absorption spectra were significantly blue-shifted at different suspension ratios. When the suspension ratio was kept constant and the dye concentration was changed, it was understood that the tendency to increase aggregation continued, but no serious absorption band shift was observed. On the other hand, the predominance of non-fluorescent H-aggregate species severely reduced the fluorescence quantum yield in cellulose nanofiber suspensions of rhodamine b molecule. Thanks to this study, it has been demonstrated that the photophysical behavior of fluorescent dyes such as rhodamine b can be adjusted in various environments, especially in nanocellulose suspensions. Thus, dyes such as rhodamine b have an important place in electronic and optoelectronic devices as photo-functional organic materials since their regulation at the molecular level can be easily controlled.

## Data Availability

No datasets were generated or analysed during the current study.
